# Dynamic ST-Segment Elevation in Massive Pulmonary Embolism Captured by Holter Electrocardiography: A Case Report

**DOI:** 10.7759/cureus.101815

**Published:** 2026-01-18

**Authors:** Ko Nagai, Taichi Kato, Seiji Domae, Kazuhiro Sugiyama

**Affiliations:** 1 Tertiary Emergency Medical Center, Tokyo Metropolitan Bokutoh Hospital, Tokyo, JPN

**Keywords:** acute pulmonary embolism, differential diagnosis in ecg, echocardiography, in-hospital cardiac arrest, point-of-care ultrasound (pocus), post-resuscitation care, st-segment elevation ecg, st-segment elevation myocardial infarction (stemi)

## Abstract

ST-segment elevation on a post-cardiac arrest electrocardiogram (ECG) is traditionally viewed as a hallmark of acute coronary occlusion. However, this finding can also arise from non-coronary etiologies like massive pulmonary embolism (PE), creating a high-stakes diagnostic dilemma. An 83-year-old woman hospitalized for trauma developed cardiac arrest with pulseless electrical activity (PEA). Immediate post-resuscitation ECG showed marked ST-segment elevation in leads V1-V4, II, III, and aVF, suggesting extensive myocardial infarction. A point-of-care echocardiogram showed severe right ventricular dilation, raising the suspicion of PE, which was confirmed by computed tomography. Fortuitously, a Holter monitor was attached, capturing the sequence of ECG changes before and after the onset of PE. It revealed that ST-segment elevation appeared after the onset of cardiac arrest and gradually decreased thereafter. The patient was successfully treated with anticoagulation. This case suggests that post-arrest ST-segment elevation in PE may reflect dynamic right ventricular strain and supply-demand mismatch, rather than fixed coronary occlusion. Recognizing the "dynamic" nature of these ECG changes, alongside rapid echocardiographic assessment, is essential to avoid unnecessary emergent coronary angiography and ensure timely treatment for PE.

## Introduction

According to major international resuscitation guidelines, ST-segment elevation on the post-resuscitation electrocardiogram (ECG) is a strong indicator of acute coronary occlusion, and immediate coronary angiography (CAG) is recommended for patients with a suspected cardiac cause of arrest. This strategy is critical for improving outcomes in patients whose cardiac arrest is caused by ST-segment elevation myocardial infarction (STEMI) [1,2]. 

However, the diagnostic accuracy of ST-segment elevation on the post-resuscitation ECG is known to be limited. Notably, recent evidence suggests that ECGs obtained within the first seven minutes after return of spontaneous circulation (ROSC) have a high false-positive rate for STEMI, approaching 18.5% [3]. Performing an emergency CAG requires the mobilization of a catheterization team and patient transport, which consumes valuable time. If the underlying cause of ST-segment elevation is not an acute coronary syndrome (ACS), this can lead to a critical delay in administering definitive therapy for the true underlying pathology. Such non-coronary causes of ST-segment elevation include metabolic derangements, epinephrine administration, and life-threatening conditions such as acute aortic dissection, intracranial hemorrhage, and massive pulmonary embolism (PE). Therefore, the decision to proceed with immediate CAG can be a pivotal moment that profoundly impacts patient prognosis. 

Here, we report the case of a patient with cardiac arrest due to a massive PE, in whom the post-ROSC ECG showed extensive ST-segment elevation, illustrating a critical diagnostic pitfall in post-resuscitation care.

## Case presentation

History and initial admission

**Figure 1:**
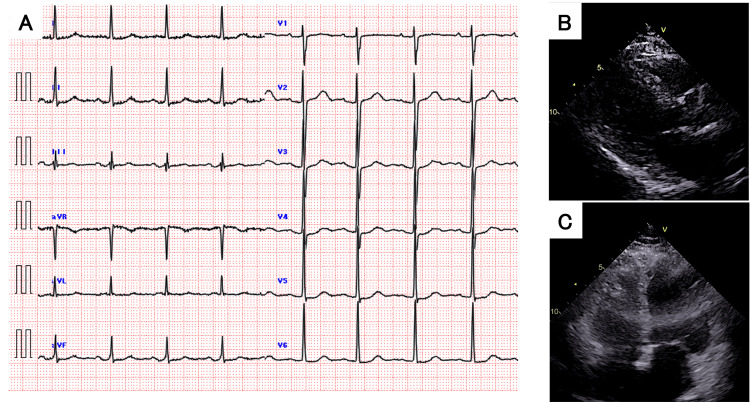
Admission ECG and echocardiogram (A) 12-lead ECG on admission showing normal sinus rhythm. (B) Parasternal long-axis and (C) apical four-chamber views on echocardiography showing normal ventricular size and function. ECG: electrocardiogram

An 83-year-old woman with a history of hypertension was transported to our hospital after a fall. On arrival, her vitals were as follows: Glasgow Coma Scale [4] 15 (E4V5M6), heart rate 84 bpm, blood pressure 150/80 mmHg, respiratory rate 16 breaths/min, and SpO2 96% on room air. A head computed tomography (CT) scan revealed traumatic acute epidural hematoma with a maximum thickness of 7 mm, leading to admission to the Department of Neurosurgery. She also sustained fractures of the right zygoma, orbital floor, and distal radius. The admission ECG and transthoracic echocardiogram showed no significant abnormalities (Figure 1). A follow-up head CT three hours later showed no expansion of the hematoma, and she was managed conservatively. The following day, a repeat CT confirmed no bleeding, and she became ambulatory.

In-hospital cardiac arrest

On hospital day 6, the patient collapsed immediately after returning to her room from the restroom. The medical emergency team responded. The initial rhythm was pulseless electrical activity (PEA). After four minutes of cardiopulmonary resuscitation (CPR), ROSC was achieved. Due to the rapid ROSC and the lack of initial intravenous access at the onset of collapse, epinephrine was not administered. However, her hemodynamics were unstable, and she remained comatose.

**Figure 2:**
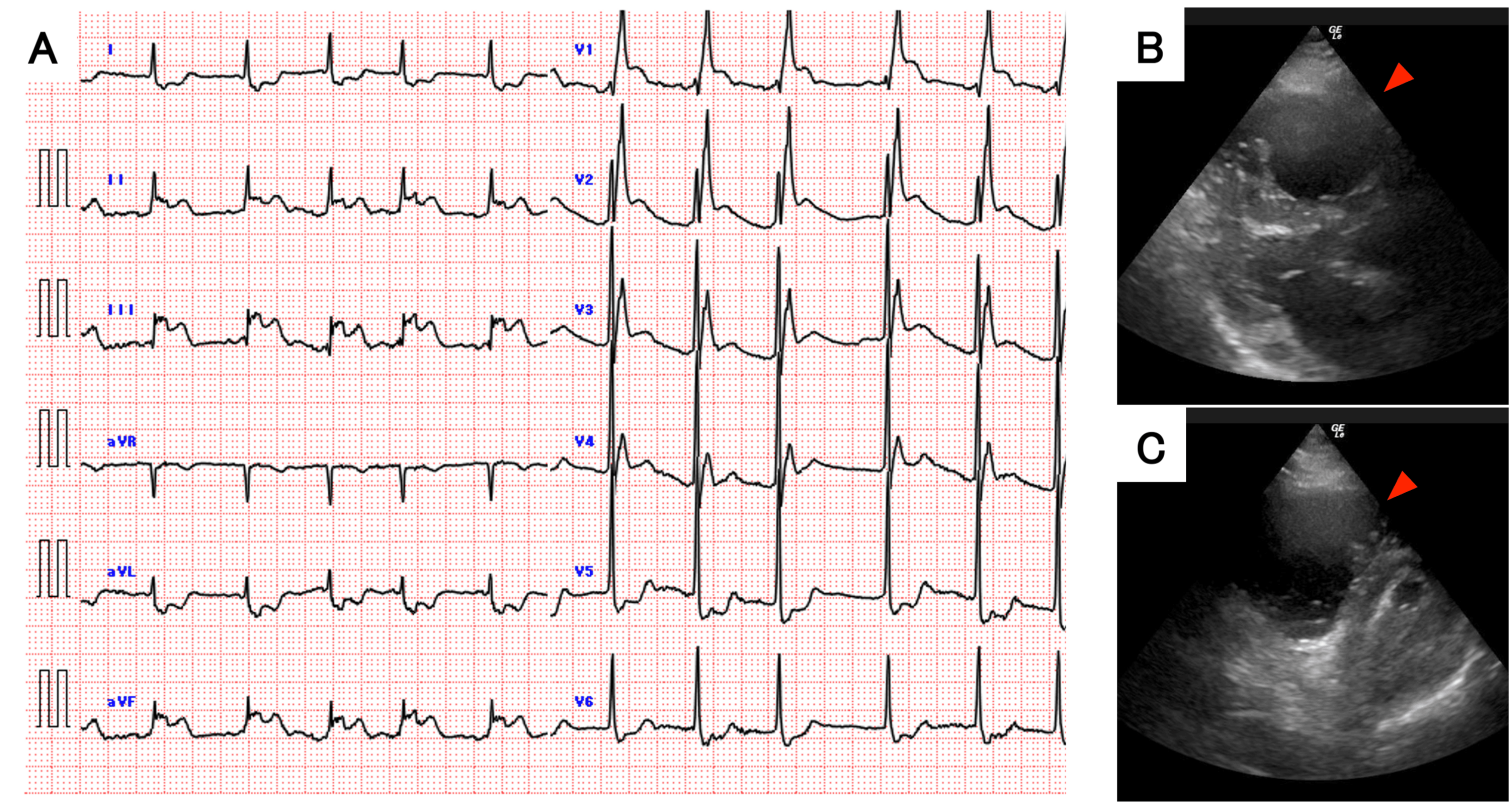
Post-resuscitation ECG and echocardiogram (A) 12-lead ECG immediately after ROSC (6 minutes post-ROSC; 10 minutes after PEA onset) showing new right bundle branch block and ST-segment elevation in leads V1-V4 and II, III, aVF. (B) Parasternal long-axis and (C) short-axis views on echocardiography showing severe right ventricular dilation and septal flattening (D-shape). The red arrows point to the enlarged right ventricle. ECG: electrocardiogram; PEA: pulseless electrical activity; ROSC: return of spontaneous circulation

A 12-lead ECG obtained immediately post-ROSC revealed a new complete right bundle branch block (RBBB) with marked ST-segment elevation in leads II, III, aVF, and V1-V4 (Figure 2A). Concurrently, point-of-care echocardiography demonstrated severe right ventricular (RV) dilation and interventricular septal flattening, forming a "D-shaped" left ventricle in the short-axis view (Figure 2B-2C). Nursing staff reported that the patient had not complained of chest pain prior to losing consciousness.

Diagnosis and outcome

Although the ECG findings suggested the possibility of STEMI, the clinical presentation of sudden PEA arrest and, most importantly, the echocardiographic evidence of severe acute RV strain strongly suggested a diagnosis of massive PE. Therefore, we prioritized contrast-enhanced CT over immediate CAG.

**Figure 3:**
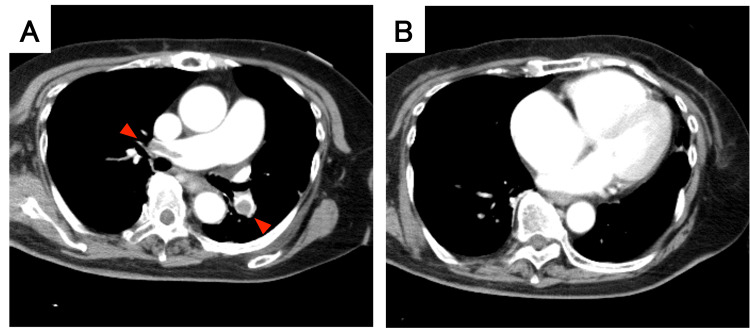
CT pulmonary angiography CT showing pulmonary embolism. (A) Emboli are observed in the bilateral pulmonary arteries (red arrows). (B) Severe right ventricular enlargement is evident. No segmental left ventricular perfusion defects were observed. CT: computed tomography

CT pulmonary angiography revealed extensive emboli in the bilateral main, lobar, and segmental pulmonary arteries (Figure 3). The scan also confirmed no perfusion defects in the left ventricular myocardium and excluded new intracranial hemorrhage or aortic dissection. Furthermore, a thrombus was identified in the right femoral vein.

Given the recent head trauma, systemic thrombolysis was contraindicated due to the high risk of intracranial hemorrhage. Anticoagulation therapy with continuous intravenous infusion of unfractionated heparin was initiated.

**Figure 4:**
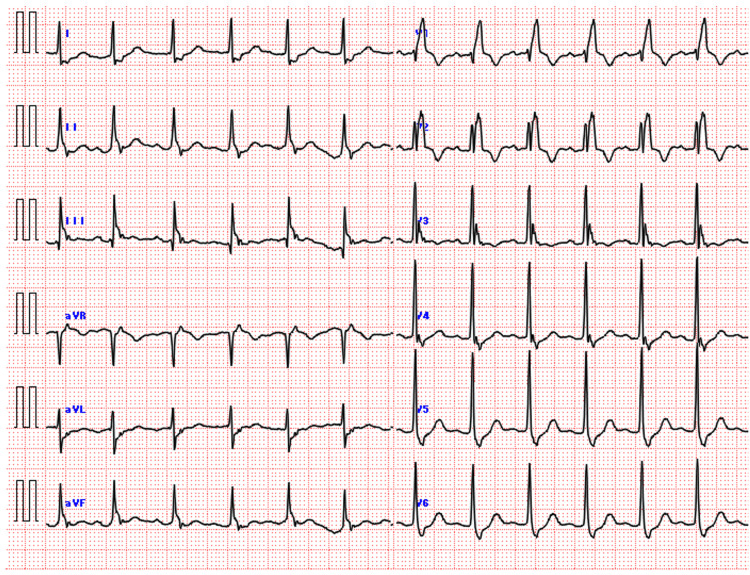
12-lead ECG one hour after ROSC The ST-segment elevation has mostly resolved, but the right bundle branch block persists. ECG: electrocardiogram; ROSC: return of spontaneous circulation

**Figure 5:**
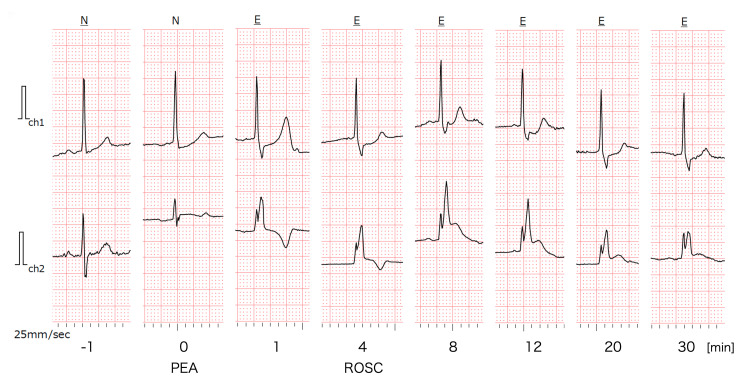
Holter ECG recording Ch1 corresponds to lead CM5 (manubrium to the left anterior axillary line, fifth intercostal space; approximating lead V5), and Ch2 corresponds to the NASA lead (manubrium to the xiphoid process; approximating lead V1). Time 0 was defined as the estimated onset of PEA, at which point the heart rate was 20 bpm. Chest compressions were performed from one to four minutes. At four minutes, compressions were discontinued, and ROSC was confirmed with a heart rate of 40 bpm. In Ch2, ST-segment began to rise during chest compressions and reached a peak of 0.5 mV at eight minutes, followed by a trend toward improvement. ECG: electrocardiogram; PEA: pulseless electrical activity; ROSC: return of spontaneous circulation

ST-segment elevation appeared during resuscitation and resolved within approximately one hour post-ROSC, but the RBBB persisted (Figure 4). Notably, a Holter monitor, which had been attached to evaluate the initial fall, provided a continuous recording of this dynamic change (Figure 5). It revealed that ST-segment elevation began during chest compressions, peaked around eight minutes after onset, and gradually improved over 30 minutes.

**Figure 6:**
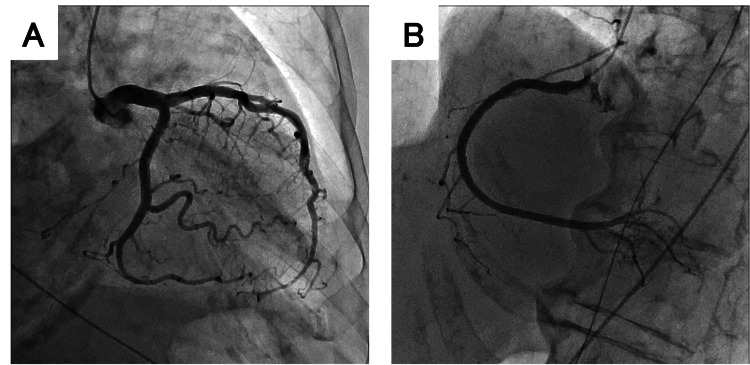
Coronary angiography Coronary angiography revealing no significant stenosis: (A) left coronary artery and (B) right coronary artery

Later on the same day, an elective CAG confirmed no significant coronary artery disease (Figure 6), supporting the diagnosis of non-occlusive myocardial ischemia.

The patient was managed in the intensive care unit (ICU) with mechanical ventilation. Her respiratory and hemodynamic status gradually improved, and she regained consciousness. The RBBB also resolved. She was discharged from the ICU on day 17 and transferred to a rehabilitation facility on day 35 with a favorable neurological outcome.

## Discussion

International resuscitation guidelines strongly recommend immediate CAG post-resuscitation if ST-segment elevation is present and a cardiac ischemic cause is suspected [1,2]. In the present case, the immediate post-resuscitation ECG displayed widespread ST-segment elevation in leads V1-V4, II, III, and aVF. Initially, acute myocardial infarction was suspected, but the distribution of ST-segment elevation was atypical. Specifically, the simultaneous ST-segment elevation in the inferior and anteroseptal territories was inconsistent with a typical single-vessel occlusion, making it difficult to explain the findings without assuming multiple coronary embolisms. While obstruction of a "wrap-around" left anterior descending artery was considered [5], the absence of ST-segment elevation in leads V5 and V6 was inconsistent with this pattern. In addition, the initial cardiac arrest rhythm was PEA. Since PEA is less commonly associated with STEMI compared to shockable rhythms, this combination of atypical ST-segment elevation and PEA suggested an alternative diagnosis. The contrast-enhanced CT revealed no obvious myocardial perfusion defects and confirmed the diagnosis of massive PE. Crucially, the ST-segment elevation resolved after the CT scan. Given this alternative diagnosis and the low probability of organic coronary occlusion, we prioritized hemodynamic stabilization in the ICU and withheld immediate CAG.

The most distinct feature of this case is the continuous ECG recording via the Holter monitor, which captured the exact onset and evolution of the ST-segment elevation (Figure 5). In most reported cases of PE mimicking STEMI, clinicians rely on a "snapshot" 12-lead ECG, leaving the precise timing of ST-segment changes uncertain. Uniquely, our Holter data demonstrated that the ST-segment elevation was not present prior to the event but began to rise concomitant with the cardiac arrest and chest compressions, peaking approximately eight minutes after onset.

This temporal correlation provides valuable physiological insight into the mechanism of ST-segment elevation in massive PE. While ST-segment elevation in acute PE is rare, it is hypothesized that a rapid increase in RV pressure and a decrease in cardiac output reduce blood flow in the right coronary artery or septal branches [6,7]. Our Holter recording supports the hypothesis of a functional etiology driven by a severe supply-demand mismatch. The rapid rise in ST segments during the low-flow state of arrest, followed by their gradual resolution, suggests that the ischemia was dependent on the immediate hemodynamic state and acute RV dilation.

However, even in acute PE, concomitant coronary artery occlusion due to paradoxical embolism or coexisting coronary artery stenosis can occur [7,8]. Therefore, although the ST-segment elevation resolved with hemodynamic stabilization, we performed elective CAG to definitively rule out these organic coronary lesions. 

Importantly, the "dynamic" nature of the ST-segment changes observed in this case serves as a crucial diagnostic clue. Unlike typical STEMI caused by plaque rupture, where ST-segment elevation generally persists until reperfusion, the ST-segment elevation in this case fluctuated according to hemodynamic status. Given the dynamic and often misleading nature of post-resuscitation ECGs and the critical time sensitivity of post-arrest care, clinicians should not rely on ECG findings alone. Instead, rapid point-of-care echocardiography plays a central role in detecting wall motion abnormalities and RV strain that differentiate PE from pure ACS [9,10], which is essential to swiftly determine the appropriate treatment strategy.

Finally, our inferences are limited by the single-case nature of this report and the lack of continuous invasive hemodynamic measurements to directly correlate with the ECG changes.

## Conclusions

This case demonstrates that post-resuscitation ST-segment elevation is not specific to acute coronary occlusion. Our unique Holter recording supports the concept that massive PE can cause transient, dynamic ST-segment changes driven by severe RV strain. A multimodal approach, prioritizing immediate point-of-care echocardiography, is indispensable for differentiating these mimics and guiding life-saving management.
